# Beyond hero and villain narratives in ecology and conservation science

**DOI:** 10.1093/biosci/biaf085

**Published:** 2025-06-30

**Authors:** Kristy M Ferraro, G Adam Meyer

**Affiliations:** Department of Biology, Memorial University of Newfoundland, St. John's, Newfoundland, Canada; Department of Biology, Memorial University of Newfoundland, St. John's, Newfoundland, Canada

**Keywords:** bias, **c**onservation biology, conservation ethics, ecology, science writing

## Abstract

Storytelling is an essential part of science writing. To craft compelling stories, scientists are taught to think of their variables as characters. A common narrative tool within ecology and conservation writing is the hero–villain trope, where a heroic protagonist faces off against an antagonistic villain. Although it is an evocative structure, we argue that this narrative structure inherently assigns moral blame to the “villains,” oversimplifies complex ecological interactions and processes, and embeds subjective values into the narrative. We then provide several solutions, including ways to deploy the hero–villain trope correctly and effectively, as well as other narrative tools that can be used in ecology and conservation writing. In fostering a more intentional approach to narrative construction, we aim to elevate the stories we tell about the natural world.

In science writing, crafting a compelling story is as important as the data itself. An engaging story commands attention. It also effectively conveys the inspiration behind research, amplifies impact, and enhances clarity, making discoveries more memorable and useful to scientists and the public (Schimel 2012). To create gripping stories, scientists are taught to treat variables as characters (e.g., Schimel 2012, Heard 2022). By casting molecules, processes, or concepts as characters, science writers can transform otherwise dry and technical information into a narrative. However, crafting an engaging story while maintaining an objective interpretation of scientific results is difficult in practice. This challenge is compounded by the growing pressure to sell one's work in an increasingly competitive scientific landscape.

Framing variables as heroes and villains is a compelling and common narrative structure in ecology and conservation science. In this device, the hero, a protagonist striving to overcome a challenge, is opposed by the villain, an antagonist that creates obstacles or threats. Presenting variables as archetypal characters is an intuitive and emotionally resonant framework that presents complex and novel scientific scenarios within a familiar schema.

However, the hero–villain trope is not without dangers to the uncareful writer. Narrative structure is both a method of information delivery and a meaningful element that shapes how readers absorb and interpret content. As such, science writers have a responsibility to critically reflect on the narrative tools we employ when communicating our science both to other experts and to the public. This includes the characterization of the entities we study.

In science writing, the hero–villain narrative structure frequently appears in three key areas: the title, the opening paragraphs that set up the paper's tensions, and/or the language used to describe key players throughout the paper. In ecological and conservation science specifically, the hero–villain framing is commonly applied to topics such as human–wildlife conflict (including resource competition with humans), predator–prey and parasite dynamics, introduced or invasive species ecology, implications of overabundance, “pest” management, and range expansion (table 1), although it exists in other topics as well. We explore the pitfalls of this narrative device for communicating scientific results in ecology and conservation science. Specifically, we argue that this narrative device can be problematic, because it inherently assigns moral blame to the villains, oversimplifies complex ecological interactions and processes, and embeds subjective values into the narrative. We then offer several strategies to deploy this trope responsibly and present alternative narrative structures that can help science writers maintain engagement without compromising scientific integrity. By fostering a more nuanced and intentional approach to narrative construction, we aim to improve both the quality and the impact of the stories we tell in scientific discourse.

**Table 1.** Examples of the hero–villain narrative in ecology and conservation.

Ecological or conservation theme	Putative examples of the narrative told through the hero–villain trope	Examples of the hero–villain trope in ecology and conservation literature
Human–wildlife conflict	Wild boars are often depicted as villains that destroy human crops.	Beasley and Rhodes 2008, Campbell and Long 2009, Thrower 2009, Moore 2010, Amici et al. 2012, Alexander and Draper 2021, Mayer et al. 2023
Overabundance	Overabundant deer are often described as invaders that damage forest systems.	Anderson 1997, Bino et al. 2010, Chee and Wintle 2010, Koons et al. 2014, Krausman et al. 2014, Byers et al. 2019, Harada et al. 2020, Spake et al. 2020, Dickie et al. 2024
Predators and parasites	Parasites are often depicted as villains that “attack” host species.	Arlian et al. 1995, Schmidt and Timm 2007, Watari et al. 2008, Russell et al. 2015, Menezes and Marini 2017, Fleming et al. 2021, Gan and Yek 2023, Torres-Romero et al. 2023, Thieltges et al. 2024
“Pest” species and outbreaks	The mountain pine beetle has been described as a threat that could bring “tragic” devastation to forest stands.	Logan and Powell 2001, Reitz 2009, Harris et al. 2016, Ivantsova et al. 2019, Stukenholtz et al. 2019, Hudgins et al. 2024, Johnstone et al. 2024, Saunier et al. 2024, Wolz et al. 2024
Nonnative species	Cane toads are often depicted as villains to native Australian predators.	Dearborn 1910, Miller and Edwards 1983, Relva et al. 2010, Blattmann et al. 2013, Krull et al. 2013, Shine 2014, Woinarski et al. 2017, Hegel et al. 2019, Rathore 2020, Suzuki and Ikeda 2020, Yang et al. 2023, Barney et al. 2024, Hudgins et al. 2024, Kinsley et al. 2024, Zhu et al. 2024, Chan et al. 2025
Range expansion	Eastern red cedar range expansion is often portrayed as devastating to grazers.	Waithman et al. 1999, Crozier et al. 2006, Andonian et al. 2011, Dawe and Boutin 2016, Holm et al. 2016, Krug et al. 2017, Sonenshine 2018, Schnelle 2023
Human actors	Humans have been described as a cancer to the earth.	MacDougall 1996, Hecht and Cockburn 2010, Moore 2010, Harte and Ehrlich 2011

*Note:* The second column lists putative examples of systems or species commonly described using the hero–villain trope, whereas the third provides references that illustrate the use of hero–villain framing through titles, introductions, or key terminology.

### The hero–villain trope assigns moral blame to villains

By definition, villains consciously and intentionally do bad things. As a result, villains are judged and held morally accountable for their deeds. Therefore, information on the morality and responsibility of characters is unavoidably built into the hero–villain trope: The villain is not only doing bad but is morally bad.

Villains and our feelings about them are human constructs. When applied to ecological and conservation writing, this narrative device risks assigning moral blame and responsibility to ecological agents and processes that cannot be held morally accountable for their actions or the outcomes of their activities (Pluhar 1988, Johannsen 2019). For example, nonhuman ecological agents, regardless of their native status, trophic level, or impact on ecological processes, act on the basis of biological imperatives and, in some cases, internal individual, social, and cultural influences (Bekoff 2010, Ferraro et al. 2023, Wooster et al. 2024). This is not to say they do not have agency that ought to be considered in ecology and conservation (Edelblutte et al. 2023), but they do not act with ill intent or make decisions shaped by human-created moral frameworks. Philosophers often classify such agents as *moral patients*. Moral patients can be considered within human moral frameworks (e.g., we may owe them some moral consideration), but they are not morally responsible for their actions and decisions. Even in cases where certain species, such as elephants, may display their own moral codes (Bekoff 2004), assigning them responsibility within a human moral framework is inappropriate (Johannsen 2019). Similarly, ecological processes cannot act with intent; a wildfire does not choose to burn a forest out of spite or anger. Although the consequences of such processes can be devastating or beneficial, depending on the perspective and context, attributing intention or moral blame to natural phenomena misrepresents their underlying mechanisms or ability to participate in human-constructed moral frameworks. Therefore, because nonhuman entities—including animals, plants, microbes, ecosystems, and even abstract forces such as climate change—cannot possess the conscious intent required to fulfill this role, the role of a villain in nonfiction writing, if used at all, must be exclusively assigned to humans. However, as we will explore later, even casting humans as villains in ecological and conservation science writing is potentially reductionist and morally problematic.

Because nonhuman ecological agents or processes do not make moral decisions, labeling them as villains risks unfairly assigning moral blame for the outcomes of ecosystem interactions or processes. For example, introduced predators in Aotearoa New Zealand are often overtly vilified in scientific writing (e.g., Potts 2009, Reardon et al. 2012, Major 2024), pitted against heroic portrayals of native flightless birds or lizards (e.g., Towns and Broome 2003, Reardon et al. 2012, Russell et al. 2015, King 2019). This narrative implies that the predators are intentionally inflicting pain on other fauna. However, the predators do not act with malice; they are simply finding a food source. Therefore, although the consequences of the predators’ actions may not align with conservation goals or the values of the people of Aotearoa New Zealand, the predators are not morally responsible for their actions. This distinction is important, because we often treat those who are morally responsible differently than we treat those who are not.

### The hero–villain is reductionist

In ecology and conservation science, the tendency to pit a “good” character against a “bad” character within an ecosystem is tempting. This narrative structure is an easy and clear way to describe ecological interactions, where one character often benefits while another is disadvantaged. However, ecosystems are made up of many interacting species, and rarely does a single antagonist or protagonist determine the success or failure of another. Reducing ecological processes to a simple hero–villain narrative oversimplifies complex dynamics. The decline of caribou, for example, is influenced by a complex interplay of factors, including habitat loss, climate change, human activity, and predator–prey dynamics (Latham et al. 2011). However, many science narratives pit heroic caribou against villainous white-tailed deer, which is depicted as “invading” caribou habitat and bringing along predators (table 1). This framing is not only misleading (white-tailed deer are not intentionally causing the caribou's decline) but also oversimplifies the ecosystem's complexity, ignoring the broader suite of factors at play (Latham et al. 2011).

Reducing ecological stories to a battle between heroes and villains can also obscure the role of humans in shaping ecosystems. For example, introduced species such as mongoose and feral cats are often villainized for their impact on native populations, as well as ecosystem functioning, regardless of their actual effects (Pereyra et al. 2024a). Similarly, introduced plants are often characterized as villains harming native plant species (e.g., Rathore 2020). But this framing rarely acknowledges the human activities that facilitated their introduction and spread (Steer 2015). For example, Towns and Broome (2003) begin their narrative with “In about 1962, ship rats invaded Big South Cape Island off southern New Zealand.” By shifting responsibility away from human agency, this oversimplification not only misrepresents the causes of ecological change but also overlooks the complexity of conservation challenges, including the role of humans in both driving and addressing these shifts (Artelle et al. 2024).

Using a hero–villain narrative to depict people or communities in ecological and conservation stories is similarly common and problematic (Bratman 2011). Although humans have the capabilities to be villains or heroes (i.e., humans are moral agents with moral responsibilities), this narrative technique often reduces the complexity of relationships and interactions to overly simplistic moral judgments (Bratman 2011). Humans are frequently portrayed as heroes when engaging in conservation efforts such as rescuing endangered species or protecting pristine wilderness. Simultaneously, humans are villainized for activities such as deforestation, poaching, or habitat destruction (table 1). Although these portrayals may reflect some truths, they obscure the nuanced realities of socioecological dynamics (Bratman 2011). In some cases, the exploitation of natural resources arises out of necessity, driven by the need to sustain communities or a lack of alternative livelihoods. Conversely, conservation efforts sometimes disregard local livelihoods or perpetuate inequalities (Dickman 2010, Brockington 2015, Thomas 2022). Humans can also be part of conservation solutions—creating just solutions to environmental problems (Zamboni et al. 2017, Tan 2021). However, by framing human individuals and communities as either heroes or villains, this trope fails to account for the complex cultural, economic, and ecological factors that influence these interactions, creating a reductionist narrative and perpetuating biases.

### The hero–villain is inherently value laden

Conservation science is inherently value driven (Soulé 1985, Odenbaugh 2003, Ferraro et al. 2021), aiming to influence policy, inform management decisions, and address societal goals (Robinson 2006, Ferraro et al. 2021). For example, decisions about which species to conserve, restoration targets, and management approaches are all guided by cultural, ethical, and economic considerations (Barry and Oelschlaeger 1996, Manfredo et al. 2021, Nelson 2021). Although ecology, as a basic science, is theoretically value neutral (Betz 2013), in practice, it is also shaped by institutional, national, and personal values (Hicks 2014, Nelson 2021). That values are embedded within conservation science or even ecology is not necessarily bad; values play a crucial role in shaping how findings are interpreted, guiding the application of knowledge and influencing future research priorities (Noss 2007). However, the values within ecology and conservation science are not always critically examined (Nelson et al. 2021, Ferraro et al. 2023). This lack of scrutiny can lead to unintended biases.

The classic hero–villain trope carries inherent value judgments, presenting “good actors” and “bad actors” in a struggle that makes a compelling narrative. When this trope is used in science writing, the implicit value judgments ascribed to the characters can become entangled with scientific conclusions in unintended and unexamined ways. Because ecological and conservation values are often deeply ingrained (Nelson et al. 2021, Ferraro et al. 2023), these fields may be especially susceptible to the unexamined influence of such narrative structures.

For example, predators and parasites are famously villainized in Western storytelling and modern sentiment (e.g., *Peter and the Wolf*, the wolf in *Little Red Riding Hood*, the python in *The Jungle Book*, and the Xenomorph in *Alien*). This characterization can be found in scientific discourse as well, particularly in the case of introduced predators (table 1). For instance in Australia, introduced feral cats and foxes are often portrayed as villains pitted against the country's “gentle and highly specialized creatures” (Troughton 1938). This framing has likely helped entrench expert opinion that these predators are key drivers of native species extinctions (Troughton 1938, Rolls 1969). However, recent research challenges this narrative, showing limited evidence to support the claim: 43.6% of extinctions have not been confirmed to have happened after the arrival of foxes, and 19.6% have not been confirmed to have happened after the arrival of cats (Wallach and Lundgren 2025).

Beyond introduced predators or species characterized as “pests” or a “nuisance”, when a prey species is framed as the narrative's protagonist, predators can easily be framed as the antagonist, or villain (table 1). This villainization can have real-world consequences. By framing predators as threats, we risk reinforcing societal hostility toward their recovery and range expansion, shaping conservation policies in ways that hinder ecological understanding (Artelle et al. 2024, Pereyra et al. 2024b, Wallach and Lundgren 2025).

The hero–villain trope can also embed values within conservation and ecological narratives in ways that lack transparency. By simplifying ecological dynamics into a binary of “good” and “bad” actors, scientists may unconsciously or consciously prioritize certain species, ecosystems, or processes while obscuring the full complexity of the issues at hand (Pereyra et al. 2024b). For example, the *enemy*–*victim* terminology used in ecology to describe parasitoids and hosts embeds an ecological relationship with moral blame. The term *enemy* also precludes any potential benefits, direct or indirect, to host species, despite evidence of more complex relationships. In addition, several of the formal hypotheses within conservation science and introduced species ecology, including enemy release (Keane and Crawley 2002), novel weapons (Cox and Lima 2006), and naive prey (Callaway 2019) embed hero–villain narratives and terminologies. The trope can also be used, at times intentionally, to galvanize public support for conservation initiatives, even if it means omitting nuances (Novotny 2023). In both cases, the lack of transparency in acknowledging the values underlying this narrative structure risks presenting subjective perspectives as objective truths.

Beyond communicating science, framing scientific narratives as heroes and villains can subtly influence study design and interpretation. Unlike other biases in data collection or analysis, which are typically acknowledged and mitigated in scientific practice, biases introduced through narrative structures or language often go unexamined. This can lead to confirmation bias, in which preconceived notions influence the design, interpretation, or presentation of research (Warren et al. 2017, Pereyra et al. 2024b, Ferraro 2025). In other words, if an ecological entity is perceived as harmful, researchers may unconsciously design experiments or interpret findings in ways that reinforce this assumption. Studies on invasive species literature highlight this issue, showing that negative language and framing frequently influence the presentation and interpretation of results (Warren et al. 2017, Mattingly et al. 2020, Wallach and Lundgren 2025). In some cases, negative language is used even when introduced species have neutral or positive effects (Pereyra et al. 2024b).

Whether or not there should be values embedded in ecology, like any other basic science, is beyond the scope of this article (Betz 2013, Hicks 2014, Nelson 2021). Similarly, we do not attempt to assess the particular values currently underpinning conservation science and practice. However, it is important to clarify that values cannot—and, arguably, should not—be entirely removed from conservation science (Barry and Oelschlaeger 1996, Odenbaugh 2003, Ferraro et al. 2023). Yet to ensure integrity, these values—including those expressed through narratives—must be transparent and intentional. The danger lies in the unintentional entanglement of values with scientific evidence, which can distort data interpretation, influence future study designs, and affect policy decisions (Pietrzyk-Kaszyńska and Olszańska 2024).

## Solutions

Narrative structure plays a powerful role in shaping how the public receives and interprets scientific findings. Scientists, by the same token, do not exist in a cultural vacuum. Rather, scientists are raised and trained in media cultures that commonly characterize animals along a hero–villain spectrum (Herzog and Galvin 1992, Geest et al. 2022, Schiffner 2023) or suggest subtle moral values, such as acceptable norms of animal harm (Stanton 2021). In conservation science—where pressing environmental and climate crises demand urgent action—questions of causality, responsibility, and redress are unavoidable. Embedded in these discussions are the fundamental questions: Who caused the problem, and who will solve it? (Chua and Schreer 2024). It is not surprising, then, that the hero–villain trope is pervasive, influencing, and being influenced by public discourse, media representation, and policy decisions (Chua and Schreer 2024, Pietrzyk-Kaszyńska and Olszańska 2024). Given the significant impact of narrative framing, scientists must be especially mindful of how they communicate their research. The way scientific findings are presented can shape not only public understanding but also conservation priorities, funding allocations, and legislative outcomes.

### Choose wisely and check thyself

Writing a good story that avoids misplaced moral blame, embedded values, and oversimplification is challenging and requires practice. Writers must approach their narratives thoughtfully and critically reflect on the story that is being told. We should ask ourselves: Have I created or perpetuated a villain? What values are embedded in my narrative? Have I been intentional and transparent about the values and roles assigned to the characters in my story?

We have argued that humans are the only entities that can be appropriately characterized as villains in ecology and conservation science. However, when a writer considers positioning either individual humans or human communities as villains, it is still crucial to consider whether this approach is the most effective and accurate way to convey the scientific story. Writers should reflect on whether the narrative would be better served by portraying humans as complex characters with nuanced roles, rather than as unequivocal antagonists or model heroes (see the “Embrace complex characters” section below).

Recognizing the narrative structure one is employing is not always straightforward, because the hero–villain trope may be either overtly stated or subtly embedded in the language and framing of the story. To critically assess the tropes being employed in one's own writing, writers should consider how they would present the story in a verbal format, such as a lecture or presentation. Reflection on how characters are introduced, the contexts in which their actions are described, and the language used to frame their roles can help identify unintended storylines, biases, or oversimplifications (Nelson et al. 2021). This can be particularly challenging as splashy titles or easy-to-digest stories make communicating science more compelling. For example, the title “Invasive Predators in New Zealand: Disaster on Four Small Paws” (King 2019) is eye-catching but primes the reader to see the predators as a villain. Relatedly, the use of analogies, similes, and metaphors within narrative arcs must be carefully considered, because they carry the same risks of obscuring truth, reinforcing biases, and influencing forms (Pereyra et al. 2024b). In addition, one can seek feedback from friendly reviewers who can explicitly evaluate these aspects. These critical steps of self-assessment and peer assessment are essential to ensure that ecological writing does not inadvertently perpetuate misleading or ethically problematic narratives. We contend that these reflections on narrative are analogous and complementary to the awareness and intentionality with which scientists employ theoretical frameworks, sampling protocols, and statistical analyses. As such, we see great value in introducing greater discussion of narrative tools in scientific training.

### Embrace complex characters

One way to avoid the pitfalls of the hero–villain trope in ecology and conservation science writing is to embrace complex characters, recognizing that ecological agents and processes often have multiple, sometimes conflicting roles in ecosystems. For instance, the introduction of a species that might easily be labeled as “invasive villains” can alter the habitat or provide a food source such that one population declines while another benefits. Zebra mussels, for example, outcompete native mussels (Burlakova et al. 2014) but also serve as an abundant food source for native ducks (Hamilton et al. 1994). Relatedly, at times the same action creates different outcomes depending on environmental context, such as when the same livestock-induced shift in plant communities accelerates soil functioning in wet sites but slows soil functioning in dry sites (Semmartin et al. 2004). Human actors also often occupy dual roles: Land managers who clear forests for agriculture may also engage in practices that enhance biodiversity on their lands. By acknowledging these complexities, ecological narratives can move away from simplistic dichotomies and instead highlight the interconnectedness and trade-offs inherent in ecosystems. This approach fosters a more nuanced understanding of conservation challenges, emphasizing that ecological processes and human actions are rarely purely good or bad but part of dynamic systems.

### Consider alternatives

Although stories with clear heroes and villains are familiar and compelling, there are other ways to engage readers with narratives that build tension and provide resolution. We describe a suite of such narrative structures, including their application to writing in ecology and conservation science (table 2). We also link each narrative structure to several cultural reference points, which we acknowledge may not be meaningful to every reader. Overall, we encourage all readers to draw inspiration from the diverse stories that inspire them. We recognize that some of the proposed narrative structures may overlap or share similarities (e.g., complex characters may appear within a *Will they, or won't they?* framework). Our intention is not to present the below narrative tools as rigid categories, but rather as flexible guideposts for more creative and nuanced storytelling in ecological and conservation science. Importantly, in using these alternatives, one must be careful not to have a character that ultimately is a villain.

**Table 2.** Examples of how a given story could be framed as either a hero–villain narrative or an alternative.

Alternative narrative structure	Example of the narrative told through the hero–villain trope	Example of the narrative told through the alternative structure
Environmental tension	Crop damage by [villain] costs agriculturalists billions of dollars and are increasing each year.	A shared environmental change places human and nonhuman actors in circumstances that challenge their traditional relationships and local coexistence.
Reckoning with the past	Overabundant [villain] is driving [hero] to extinction by dramatically altering the habitat of [hero], despite human efforts to reduce damage caused by [villain].	Because of the human-driven extinction of predators, community and population dynamics have changed in a way that requires further study or intervention.
Outsider looking in	[Villain predator or parasitoid] kills [heroic victim].	Exploration of how the interaction between a parasite and a host works, and the interaction's ecological consequences.
Will they, or won't they?	Outbreak of [villainous] “pest” would threaten harvest yield and damage industry.	Shifting industry practices and climate change could lead to more frequent outbreaks of [native insects], or, alternatively, greater stabilization of populations at low density.
Character study	Nonnative invading [villain] is driving native [hero] to population collapse, despite human efforts to reduce damage caused by [villain].	The introduction of a nonnative species has brought new roles and dynamics into the community which must be uncovered and explored.
Place-based narrative	Expanding population of [villain] is displacing local [hero], destabilizing ecosystems.	Because of climate change, the abiotic and biotic community of a given space has changed, leading to new dynamics in a place. How have these dynamics in this place changed over time?
Inconvenient prophecy	[Heroic] conservation efforts stem the tide of [villainous] deforestation.	Because of the ever-growing need for timber, conservationists must find a way to deal with deforestation.

### Character study

A popular tool in fiction is the character study, which is a writing device used to explore the complex internal dynamics of an individual or group—think *Love in the Time of Cholera* by Gabriel García Márquez or the films *Good Will Hunting* or *Phantom Thread*. In this writing device narrative tension is built via the unknown motivations or drivers of a character's observed behaviors or traits. The resolution comes when the reader gains a more fulsome, multidimensional understanding of the character; you learn about what makes the character tick, and previously mysterious behaviors or patterns are explained. In ecology or conservation, a character study approach offers a framework for presenting a descriptive analysis of an ecological entity—whether an individual organism, population, species, or process.

This method involves treating the subject as a character to be studied in depth, providing a nuanced and value-neutral account of its traits, behaviors, and ecological roles (e.g., Ballari and Barrios‐García 2014, Flemming et al. 2016, Markov et al. 2022, Hanberry and Faison 2023, Martínez-Soto and Johnson 2024). The narrative could center on the character's interactions with its environment, its contributions to ecosystem processes, or its responses to external pressures, such as climate change or human activity. For example, Flemming and colleagues (2016) assessed the multidimension roles of hyperabundant geese in arctic ecosystems, including potential conservation concerns, without creating a villain. Initially motivated by declining populations of other Arctic-breeding shore birds, the authors provide a morally neutral but compelling account of positive and negative goose–ecosystem interactions. Overall, they argue that a variety of goose effects—altered nesting habitat, prey availability, and predator–prey interactions—may indeed influence shore bird population dynamics, without implying moral culpability of geese (Flemming et al. 2016). For example, instead of stating that “geese separate chicks from their mothers by trampling through nesting sites,” the authors described that “foraging geese may flush other incubating birds from their nests at more frequent intervals causing the eggs or chicks to be left without incubation for longer periods” (Flemming et al. 2016).

Similarly, Martínez‐Soto and Johnson (2024) showed that positive effects of fiddler crab on cordgrass biomass in their historic range flip to negative effects in a climate-expanded range. They highlight potential mechanisms and concerns for future ecosystem functioning without implicating fiddler crabs as a villain, placing any moral responsibility for changes in the ecosystem on humans by highlighting the fiddler crab's status as a climate migrant (Martínez-Soto and Johnson 2024). By focusing on detailed observations and avoiding prescriptive or moral judgments, the character study can reveal complex interdependencies and highlight the intricate dynamics of ecological systems.

### Place-based narrative

Similar to a character study*,* a place-based narrative structure focuses on the description of a place and the actors, appealing to people's curiosity about how things work in different places. Historically, both popular (e.g., Thoreau 1971) and scientific (e.g., Forbes 1887, Cowles 1899) ecological writing was predominantly descriptive, capturing a snapshot of a place in time. This trend continues today in many nature documentaries (e.g., *Planet Earth* or *March of the Penguins*). This approach provides a powerful way to engage readers by immersing them within ecological systems and the mechanisms that drive them. From Cowles (1899): “Perhaps no topographic form is more unstable than a dune. [Plants] are obliged to adapt themselves to a new mode of life within years rather than centuries, the penalty for lack of adaptation being certain death.”

Beyond describing the setting, this narrative style also allows for the exploration of the complex characters within a place—be they species, communities, or ecological processes—and the intricate relationships that bind them (e.g., Lindeman 1942). This type of narrative is particularly well suited for studies in ecosystem and community ecology (e.g., Hockridge et al. 2024, Monk et al. 2024), human–wildlife coexistence (e.g., Gogoi et al. 2024), or studies of community-based conservation programs, where the emphasis can be on understanding interactions and processes (e.g., Abebe et al. 2020, Kudalkar and Veríssimo 2024). For example, Monk and colleagues (2024) immersed readers in the high Andean ecosystem of San Guillermo National Park (Argentina) to learn how many characters—vicuñas, pumas, condors, plants, and resource flows—form complex interrelations in a metaecosystem. By centering on the place and characters within itself, this narrative approach invites readers to appreciate the richness of ecological systems.

### Environmental tension

Environmental tension is a narrative device used when the setting itself becomes an active force, shaping the challenges and dynamics that unfold—think Jack London's *Call of the Wild* or the movie *Castaway.* This approach can be particularly effective in ecological and conservation writing, because it highlights the interplay between organisms and their environments. Narratives centered on an agent—whether a species, individual, or community—struggling to adapt or survive in a harsh or unfamiliar environment create compelling tension (e.g., Egli and Work 2024, Liang et al. 2024, Piper et al. 2024, Song et al. 2024). A strong example was provided by Pearse and colleagues (2024) and Ambrose and colleagues (2015), who framed extreme weather events, particularly drought, as a challenging environmental condition affecting their focal community: waterbirds and redwoods, respectively. Similarly, species reintroductions could be well suited for this narrative style, because they inherently involve friction between established systems and new elements. By emphasizing the setting as both a backdrop and a dynamic player in the story, this framework draws attention to the complexities of environmental change and adaptation, immersing readers in the stakes and consequences of ecological interactions without ascribing moral value.

### Will they, or won't they?

Narrative tension can be effectively built around uncertainty and unresolved questions—think Jane Austen's *Pride and Prejudice*, Leo Tolstoy's *Anna Karenina*, or the film *When Harry Met Sally*. Given its focus on an unresolved question, the *Will they, or won't they?* structure is a natural fit for ecological and conservation writing. For example, research on partial migration, where animals must decide whether to migrate or remain resident, lends itself well to this trope by examining the factors influencing these critical decisions (e.g., Meyer and Nelson 2019, Poole et al. 2024, Witczak et al. 2024). For example, Poole and colleagues (2024) began their narrative by outlining the many factors that influence ungulate migration, painting a picture of the complex landscape and population dynamics that drive changes in migratory behavior. They then built narrative tension by highlighting recent shifts in their study region, the East Kootenary, that may alter the movement patterns of the resident elk population. Similarly, research on animal decision-making (e.g., Johansson et al. 2024), plant responses to environmental change or perturbation (e.g., Tourville et al. 2024), and experimental manipulations testing ecological tipping points—such as studies assessing whether the Atlantic Meridional Overturning Circulation might shut down—build suspense by probing the mechanisms driving these potential outcomes. This framing can also be applied in a value-neutral way to studies on introduced species, asking whether they will significantly affect native species (Fehr et al. 2024). By centering on unresolved questions, this narrative style captivates readers, engaging them with the uncertainty and stakes of scientific discovery while highlighting the dynamic or decision-driven aspects of ecological systems.

### Outside looking in

The outside looking in narrative creates tension by exploring phenomena that are inherently inaccessible to the observer, making it particularly suited for research on animal cognition and behavior or plant function—think the film *Arrival* or *The Left Hand of Darkness* by Ursula K. Le Guin. Human attempts to understand the inner workings of nonhuman minds are, by nature, limited by our inability to directly experience or fully comprehend another species’ perspective. This creates a compelling narrative as researchers piece together evidence, using behavioral studies, comparative psychology, neurobiology, and ecological context to infer how animals perceive, think, and make decisions (Jamieson 2009). Similarly, relationships between individual actors within a system can be framed this way. For example, predator–prey dynamics can be explored to uncover how they function and the subtle tensions and balances within these relationships, rather than imposing a moral framing (e.g., Bartra-Cabré et al. 2024, Wagnon et al. 2024). This narrative style not only engages curiosity but also underscores the humility and wonder inherent in ecological research (Ferraro et al. 2023, Vucetich et al. 2025), as humans attempt to bridge the gap between our perspective and that of the other beings with whom we share the planet.

### Reckoning with the past

The reckoning with the past narrative is a tool that forces characters to deal with unresolved past events or issues—think *The Kite Runner* by Khaled Hosseini, *The Godfather Part II*, or Hiromu Arakawa's series *Fullmetal Alchemist*. The structure provides a powerful framework for ecological and conservation writing, especially when exploring humanity's profound impact on ecosystems over time. By framing an ecological study using this narrative tool, researchers can assess the legacy effects of human activity, exploring how past land-use decisions, resource exploitation, or conservation efforts have left enduring marks on our planet (e.g., Doughty et al. 2016, Lemoine et al. 2023, Witteveen et al. 2024).

This approach invites a critical examination of historical data, ecological records, and oral histories to uncover how the past shapes the present ecological reality in a way that acknowledges and mitigates historical inequities or damages. For instance, Svenning and colleagues (2024) reevaluated the evidence for human involvement in the decline of Pleistocene megafauna and exemplify this narrative style by revisiting and critically analyzing historical ecological events. Paleoecology, in particular, lends itself well to this trope, because it seeks to uncover how past human activities—from hunting to land-use changes—have shaped ecosystems and biodiversity. By reflecting on these impacts, this narrative invites readers to confront humanity's role in shaping the natural world.

Many ecological systems bear the imprint of past disturbances which are not a result of human action. Fires, floods, volcanic eruptions, and other natural events often leave lasting legacies that can cascade through ecosystems for decades or even centuries. Therefore, although reckoning with the past can be a useful structure for exploring the impacts of human-driven disturbances on ecological systems, it also provides a lens to examine how natural disturbances shape ecosystems over time.

### Inconvenient prophecy

The inconvenient prophecy narrative arc places characters into difficult choices or unforeseen challenges, often driven by unwelcome or inconvenient future scenarios—think the film *Interstellar*, *Macbeth* by William Shakespeare*,* or *The Goldfinch* by Donna Tartt*.* This structure is particularly relevant in ecology when characters—whether individuals, communities, or ecosystems—must deal with future changes in dynamics. This could include unprecedented anthropogenic climate change, land-use change, and biodiversity loss, where the looming consequences force uncomfortable reckonings (e.g., Brown et al. 2024, Gittman et al. 2024, Worthy et al. 2024). For example, Worthy and colleagues (2024) created narrative tension by describing seed germination as a trait finely tuned to specific environmental conditions. Under projected climate change scenarios, the authors describe how shifts in seasons could disrupt this timing, potentially leading to germination failure—an inconvenient prophecy grounded in ecological forecasting. But the authors remain open to either outcome, stating “understanding variation among species’ germination strategies and responses to environmental cues for germination can reveal vulnerability or resilience to climate change” (Worthy et al. 2024). Similarly, models predicting shifts in climate zones or the collapse of key ecosystems place policymakers, conservationists, and communities in positions where they must grapple with difficult trade-offs. This narrative arc can be used to highlight the urgency and complexity of ecological problems and emphasize the resilience and creativity required to navigate a future shaped by the consequences of human activity.

## Conclusions

The strength of ecological storytelling lies in its ability to inspire and inform, but this power comes with a responsibility to honestly represent the nuanced realities of ecological systems. Simplistic hero–villain narratives can distort ecological realities, obscure human culpability, and shape ecological science and conservation priorities in unintended ways. We expect that many scientists will find the reductionism and moral blame inherent to the hero–villain trope does not align with their intellectual understanding of the systems they study. By highlighting alternative narratives, we hope to inspire more nuanced and value-transparent storytelling that better reflects scientists’ deeper, more accurate understanding of ecological challenges. Moving beyond moralistic binaries allows for narratives that are not only compelling but also scientifically rigorous—better equipping us to address the complexities of a rapidly changing world.

## Notes

Kristy Ferraro (kristy.m.ferraro@gmail.com) is a Banting Postdoctoral Fellow and Adam Meyer (gameyer@mun.ca) is a PhD candidate in the Department of Biology at Memorial University of Newfoundland, in St. John's, Newfoundland, Canada.
